# Diarylheptanoid Glycosides of *Morella salicifolia* Bark

**DOI:** 10.3390/molecules22122266

**Published:** 2017-12-19

**Authors:** Edna Makule, Thomas J. Schmidt, Jörg Heilmann, Birgit Kraus

**Affiliations:** 1 Department of Pharmaceutical Biology, Faculty of Chemistry and Pharmacy, Universität Regensburg, Universitätsstraße 31, D-93053 Regensburg, Germany; edna.makule@nm-aist.ac.tz; 2 Department of Food Biotechnology and Nutritional Sciences, Nelson Mandela African Institution of Science and Technology, P.O. Box 447 Arusha, Tanzania; 3 Institute of Pharmaceutical Biology and Phytochemistry (IPBP), Westfälische Wilhelms-Universität Münster, PharmaCampus—Corrensstraße 48, D-48149 Münster, Germany; thomschm@uni-muenster.de

**Keywords:** *Morella salicifolia*, Myricaceae, diarylheptanoid glycosides, myricanol, juglanin

## Abstract

A methanolic extract of *Morella salicifolia* bark was fractionated by various chromatographic techniques yielding six previously unknown cyclic diarylheptanoids, namely, 7-hydroxymyricanol 5-*O*-β-d-glucopyranoside (**1**), juglanin B 3-*O*-β-d-glucopyranoside (**2**), 16-hydroxyjuglanin B 17-*O*-β-d-glucopyranoside (**3**), myricanone 5-*O*-β-d-gluco-pranosyl-(1→6)-*β*-d-glucopyranoside (**4**), neomyricanone 5-*O*-β-d-glucopranosyl-(1→6)-β-d-glucopyranoside (**5**), and myricanone 17-*O*-α-l-arabino-furanosyl-(1→6)-β-d-glucopyranoside (**6**), respectively, together with 10 known cyclic diarylheptanoids. The structural diversity of the diarylheptanoid pattern in *M. salicifolia* resulted from varying glycosidation at C-3, C-5, and C-17 as well as from substitution at C-11 with hydroxy, carbonyl or sulfate groups, respectively. Structure elucidation of the isolated compounds was achieved on the basis of one- and two-dimensional nuclear magnetic resonance (NMR) as well as high-resolution electrospray ionisation mass spectrometry (HR-ESI-MS) analyses. The absolute configuration of the glycosides was confirmed after hydrolysis and synthesis of *O*-(*S*)-methyl butyrated (SMB) sugar derivatives by comparison of their ^1^H-NMR data with those of reference sugars. Additionally, absolute configuration of diarylheptanoid aglycones at C-11 was determined by electronic circular dichroism (ECD) spectra simulation and comparison with experimental CD spectra after hydrolysis.

## 1. Introduction

*Morella salicifolia* (Hochst. ex A. Rich.) Verdc. & Polhill belongs to the family Myricaceae in the order Fagales. It was formerly named *Myrica salicifolia* Hochst. ex A. Rich. until the genus *Myrica* was divided into two genera: *Myrica* and *Morella. M. salicifolia* now belongs to the genus *Morella*, with the new accepted name *Morella salicifolia* (Hochst. ex A. Rich.) Verdc. & Polhill. (syn. *Myrica salicifolia* Hochst. ex A. Rich.) [1]. *M. salicifolia* is reported to be spread in many mountainous ranges in Tanzania above 1200 m and prefers shallow soil, heath, and rocky areas [2]. The species is distributed mainly in Tanzania, Kenya, Uganda, Rwanda, Burundi, Ethiopia, Democratic Republic of the Congo, Yemen, and Saudi Arabia [2,3]. Traditional medicinal use of *M. salicifolia* has been previously reported in Tanzania where it is used for the treatment of cough, toothache, decoction, tonic, stomach troubles, skin diseases [4], headaches [5], and opportunistic diseases of human immunodeficiency virus/acquired immune deficiency syndrome such as tuberculosis, chronic diarrhoea, cryptococcal meningitis, and herpes simplex [6]. Further, traditional medicinal uses of *M. salicifolia* have been reported in other countries such as Ethiopia where it is used for the treatment of skin diseases [7], pain, inflammation, and respiratory disorders [8,9]. In Uganda, *M. salicifolia* has been used for the treatment of male sexual impotence and erectile dysfunction [10]. 

Despite the many traditional uses of *M. salicifolia*, literature describing its phytochemical and pharmacological investigation is scarce. A preliminary phytochemical screening of the methanolic extracts of the stem bark and leaves showed the presence of polyphenols, unsaturated sterols/triterpenes, saponins, glycosides, and carbohydrates [11]. Moreover, results from previously conducted biological activity studies on *M. salicifolia* showed that a methanolic extract of *M. salicifolia* stem bark was effective against *Bacillus cereus*, *Neisseria gonorrhoeae*, *Shigella dysenteriae*, and *Staphylococcus aureus* [11,12]. In vivo testing of a methanolic extract of *M. salicifolia* in mice showed potent analgesic and antipyretic activity at a concentration of 100 mg/kg [13].

To date, no study has reported the isolation of individual compounds from *M. salicifolia*. Therefore, the aim of this work was the isolation and structure elucidation of secondary metabolites from crude methanolic extract of *M. salicifolia* bark to explore the documented activity and traditional usage of the drug in future studies.

## 2. Results

**Figure 1:**
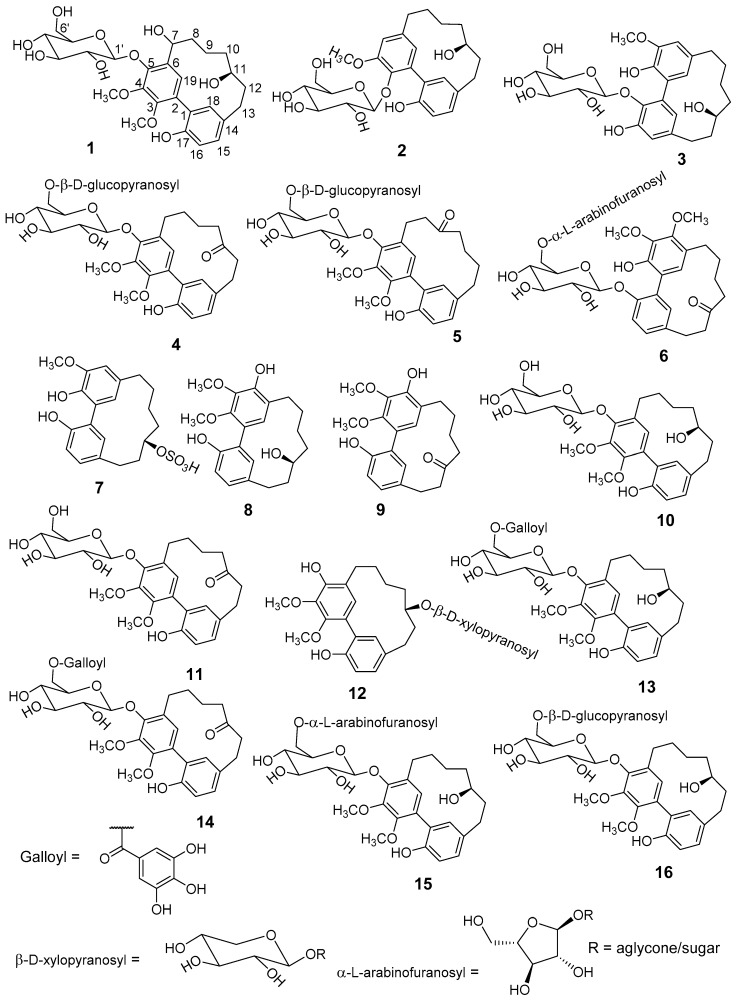
Structures of the isolated diarylheptanoids from methanolic extract of *Morella* *salicifolia* bark. Compounds **1**–**6** (new) and **7**–**16** (known).

Fractionation of a methanolic extract of *M. salicifolia* bark resulted in the isolation of 6 unknown (**1**–**6**) and 10 known (**7**–**16**) cyclic diarylheptanoids of the sub-group *meta-meta* cyclophane (Figure 1). The known diarylheptanoids were juglanin B-sulfate (**7**) [14,15], myricanol (**8**) [16,17,18,19], myricanone (**9**) [16,19], myricanol 5-*O*-β-d-glucopyranoside (**10**) [20], myricanone 5-*O*-β-d-glucopyranoside (**11**) [21], myricanol 11-*O*-β-d-xylopyranoside (**12**) [22], myricanol 5-*O*-β-d-(6’-*O*-galloyl)-glucopyranoside (**13**) [23], myricanone 5-*O*-β-d-(6’-*O*-galloyl)-glucopyranoside (**14**) [15], myricanol 5-*O*-α-l-arabinofuranosyl-(1→6)-β-d-glucopyranoside (**15**) [24], and myricanol gentiobiosidse: myricanol 5-*O*-*β*-d-glucopranosyl-(1→6)-β-d-glucopyranoside (**16**) [23], respectively. Structure elucidation was done by comprehensive one- and two-dimensional nuclear magnetic resonance (NMR), as well as high-resolution electrospray ionisation mass spectrometry (HR-ESI-MS), sugar hydrolyses, circular dichroism (CD)-analyses, and comparison with published data.

The HR-ESI-MS of compound **1** exhibited ions at *m*/*z* 535.2185 [M − H]^−^ and 1071.4445 [2M − H]^−^ which are consistent with the molecular formula C_27_H_36_O_11_. The ultraviolet (UV) spectrum (MeOH) of **1** showed absorption maxima at 213, 250, and 295 nm, which are typical for cyclic biphenyl-type diarylheptanoids [22,25]. The ^1^H-NMR spectrum of **1** was similar to published data on compound **10** [20] showing four aromatic protons resonating at δ_H_ 7.02 (1H, dd, *J* = 2.2, 8.2, H-15), δ_H_ 6.77 (1H, d, *J* = 8.2, H-16), and δ_H_ 6.87 (2H, s, H-18, H-19). Two aromatic methoxy groups were observed as one singlet at δ_H_ 3.95 (6H, H_3_-20 and H_3_-21) and were placed at C-3 and C-4 due to the [^1^H-^13^C]-heteronuclear multiple bond correlations (HMBC). Five high-field shifted methylene groups were observed as multiplets between δ_H_ 1.12 and 2.88 and assigned with the help of [^1^H-^1^H]-correlated spectroscopy (COSY) and [^1^H-^13^C]-HMBC experiments to H-8 (δ_H_ 2.13, m and δ_H_ 1.97, m), H-9 (δ_H_ 1.52, m and δ_H_ 1.15, m), H-10 (δ_H_ 1.79, m and δ_H_ 1.52, m), H-12 (δ_H_ 2.13, m and δ_H_ 1.63, m), and H-13 (δ_H_ 2.84, m). Two methane groups bearing a hydroxy group resonated at δ_H_ 3.84 (m) and 4.92 (dd, *J* = 3.6, 11.4) and were assigned to H-11 and H-7 following a proton coupling network from the COSY and HMBC long-range correlations. Sugar protons typical of a glucose resonating between δ_H_ 3.32 and 3.80 were assigned to H-2′–H-6′ based on the COSY experiment. The anomeric proton of the glucose moiety was observed at δ_H_ 5.05 (H-1′, *J* = 7.4 Hz), suggesting β-configuration. The position of the glycosidic linkage was elucidated from the HMBC long-range correlation between H-1′ and C-5 (δ_C_ 150.0). From obtained the UV, NMR (Table 1 and Table 2), and HR-MS data, compound **1** was concluded to be a hitherto undescribed diarylheptanoid, 7-hydroxymyricanol 5-*O*-β-d-glucopyranoside, and was named salicimeckol.

Compound **2**, as a second previously unknown cyclic diarylheptanoid monoglycoside, was identified as juglanin B 3-*O*-β-d-glucopyranoside and named salicireneol A. HR-ESI-MS showed ions at *m*/*z* 489.2137 [M − H]^−^ and *m*/*z* 979.4324 [2M − H]^−^ corresponding to a molecular formula of C_26_H_34_O_9._ Structure elucidation was done in analogy to compound **1** by extensive 1D and 2D NMR measurements. Accordingly, the ^1^H- and ^13^C-NMR data for **2** are in agreement with published data of juglanin B [26,27] complemented by additional sugar signals (Table 1 and Table 2). 

The molecular mass of compound **3** was deduced from HR-ESI-MS ions at *m*/*z* 505.2078 [M − H]^−^ and 551.2137 [M + HCOO]^−^ calculated for the molecular formula of C_26_H_34_O_10_. The ^1^H-NMR of **3** showed four aromatic protons, at δ_H_ 7.04 (H-5, brs), *δ*_H_ 6.87 (H-19, brs), δ_H_ 6.64 (H-18, brs), and δ_H_ 5.66 (H-15, brs). Furthermore, signals attributed to six aliphatic methylene groups were detected between δ_H_ 0.90 and 2.78 and were assigned to positions H-7–H-10, H-12, and H-13 (Table 1). One methoxy group resonated at δ_H_ 3.80 (s, 3H) and was assigned to H_3_-20 by [^1^H-^1^H]-rotating-frame nuclear Overhauser effect correlation spectroscopy (ROESY) and HMBC correlations. The anomeric proton of a β-glucopyranosyl moiety was observed at δ_H_ 5.00 (H-1’, d, *J* = 7.6 Hz) and the glycosidic linkage of **3** was elucidated by an HMBC experiment showing a long-range correlation to C-17 (δ_C_ 144.7 ppm, Table 2). The position of the hydroxy group at C-11 was deduced from COSY due to lacking long-range correlation signals of H-11 (δ_H_ 3.05, m) in the HMBC experiment. Therefore, compound **3** is 16-hydroxyjuglanin B 17-*O*-β-d-glucopyranoside, a hitherto unknown compound and named salicireneol B.

HR-ESI-MS of **4** and **5** showed ions at *m*/*z* 679.2682 [M − H]^−^ and *m*/*z* 679.2622 [M − H]^−^ with the common molecular formula of C_33_H_44_O_15_ and calculated mass of 680 Da. The ^1^H-NMR of **4** showed signals at δ_H_ 3.94 and 3.80 (both s, 3H), which were assigned to the two methoxy groups H_3_-20 and H_3_-21. Four aromatic protons were assigned to H-15 (7.05, dd, *J* = 2.1, 8.2), H-16 (6.80, d, *J* = 8.2), H-18 (6.64, brs), and H-19 (6.57, s) of the aglycone (Table 1). The carbonyl groups at δ_C_ 217.2 ppm were assigned to position C-11 as deduced from the HMBC experiment due to long-range correlations between H-9 (δ_H_ 1.70, m, 2H), H-10 (δ_H_ 2.78, m, 1H and δ_H_ 2.65, m, 1H), H-12 (δ_H_ 2.91, m, 2H), H-13 (δ_H_ 2.78, m, 1H), and the carbonyl carbon. Further confirmation was achieved from the COSY experiment due to observed cross peaks between H-7 (δ_H_ 2.91, m, 1H and δ_H_ 2.78, m, 1H) and H-8 (1.89, m, 2H), H-8 and H-9 (1.70, m, 2H), H-9 and H-10 (δ_H_ 2.78, m, 1H and δ_H_ 2.65, m, 1H), and H-12 (δ_H_ 2.91, m, 2H) and H-13 (δ_H_ 2.78, m, 1H). The anomeric protons of two glucosyl moieties resonated at δ_H_ 4.99 (d, *J* = 7.2 Hz, H-1’) and δ_H_ 4.28 (d, *J* = 7.8 Hz, H-1”). Long-range correlations were observed between the anomeric proton H-1’ and C-5 (δ_C_ 149.8 ppm) of the aglycone and the anomeric proton H-1” to C-6’ of the glucose. Therefore, two sugar groups were attached to C-5 of the aglycone and they were confirmed to be two d-glucose molecules. Hence, the structure of **4** was confirmed to be the hitherto unknown myricanone 5-*O*-β-d-glucopranosyl-(1→6)-β-d-glucopyranoside and was given the name saliciclaireone A.

The carbon data of **5** was found to be similar to the published data of neomyricanone 5-*O*-β-d-glucopyranoside [22], except that **5** was found to have two 1→6 connected glucose moieties attached to C-5. Extensive one- and two-dimensional NMR revealed that compound **5** is neomyricanone 5-*O*-β-d-glucopranosyl-(1→6)-β-d-glucopyranoside, a hitherto undescribed compound which was thus named saliciclaireone B. The summarized 1D-NMR data for **4** and **5** were depicted in Table 1 and Table 2, respectively.

The HR-ESI-MS of **6** showed a pseudomolecular ion at *m*/*z* 649.2509 [M − H]^−^ in the negative mode, consistent with the molecular formula of C_32_H_41_O_14_. The ^1^H-NMR spectrum shows 2 methoxy groups at δ_H_ 3.81 and 3.93 ppm (both s, H_3_-21 and H_3_-20), 4 aromatic protons, and 6 methylene groups. A carbonyl group resonating at δ_C_ 216.1 was assigned to C-11 based on the HMBC experiment showing long-range correlations to δ_H_ 2.80/2.61 (H-10), δ_H_ 2.93/2.79 (H-12), and δ_H_ 2.95 ppm (H-13, 2H). Two anomeric protons observed at δ_H_ 4.97 (H-1’, d, *J* = 7.2) and δ_H_ (4.83 H-1” brs), indicated the presence of two sugar moieties with a β-d and α-l-configuration. The positions of glycosidic linkages were elucidated from the HMBC experiment. Long-range correlation was observed between H-1’ (δ_H_ 4.97) and C-17 (δ_C_ 152.7) of the aglycone as well as H-1” (δ_H_ 4.83) and C-6’ (δ_C_ 68.0) of the glucose. Therefore, it was concluded that the two sugar groups are attached to C-17 of the aglycone. The NMR signals of the aglycone were similar to those of myricanone (compound **12**).

The attached glycosides were confirmed for β-d-glucopyranoside and α-l-arabinofuranoside as described in section of absolute configuration of isolated diarylheptanoids. The furan ring of the α-l-arabinose was concluded based on its carbon chemical shifts as described by Beier and Mundy [28]. Based on the obtained data, compound **6** was identified to be myricanone 17-*O*-α-l-arabinofuranosyl-(1→6)-β-d-glucopyranoside and was named saliciclaireone C. The 1D-NMR data of compound **6** are summarized in Table 1 and Table 2. ^1^H-NMR of compounds **1**–**6** can be found in the Supplementary Materials.

**Figure 2:**
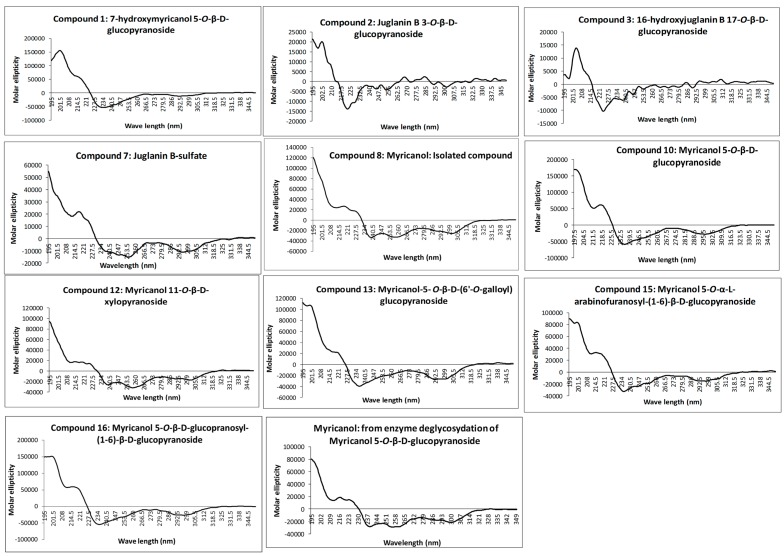
Recorded experimental circular dichroism (CD) spectra of isolated diarylheptanoids (molar ellipticity vs. wavelength. (nm)).

The absolute configuration at C-11 was determined as 11*R* for all isolated diarylheptanoids by measurement of the CD spectra of the aglycone and comparison with electronic CD spectra simulation. Recorded experimental CD spectra of the isolated diarylheptanoids and the aglycone obtained from enzyme hydrolysis were found to be very similar (Figure 2) and it was hence concluded that the attached glycosides and sulfate moieties had no influence on conformation of the aglycone and its chromophore. Therefore, CD spectra simulation was performed using myricanol as a model compound. The myricanol structure contains one chiral center at C-11, which is axially dissymmetric due to the twisted biphenyl. Thus, the structure of myricanol can occur as two pairs of enantiomers i.e., (*R*,*Ra*), (*S*,*Sa*), and (*R*,*Sa*), (*S*,*Ra*), where “a” stands for the chirality of the axially dissymmetric biphenylic system [18,29].

**Figure 3:**
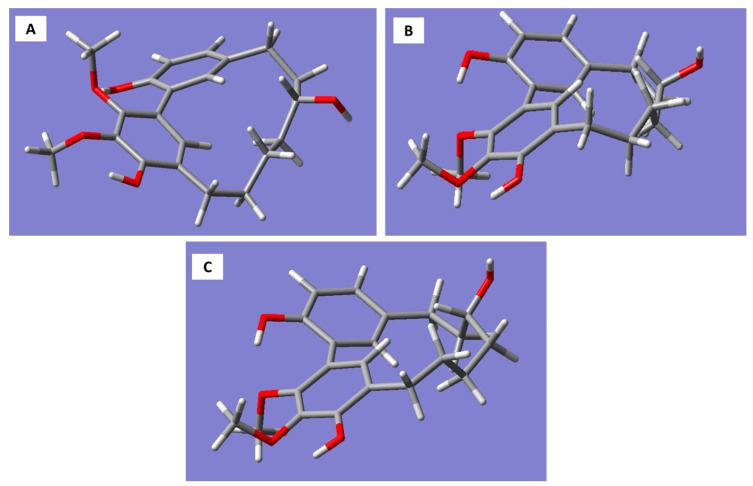
Three-dimensional structures of the conformations of *R-*myricanol. (**A**) 11*R*,*Ra* myricanol. RB3LYP/6-31G(d,p) energy: –1192.01521911 a.u. Energy difference from lowest conformer: 2.01 kcal/mol; (**B**) 11*R*,*Sa* myricanol (a). RB3LYP/6-31G(d,p) energy: −1192.01342066 a.u. Energy difference from the lowest conformer: 3.31 kcal/mol; (**C**) 11*R*,*Sa* myricanol (b). Conformation corresponds to the X-ray crystal structure [19]. RB3LYP/6-31G(d,p) energy: –1192.01842217 a.u. Energy difference from the lowest conformer: 0.000 kcal/mol.

Molecular models generated for the *R*-enantiomer of myricanol resulted in three conformations corresponding to the 11*R*,*Ra*, 11*R*,*Sa* (a), and 11*R*,*Sa* (b) forms (Figure 3A–C).

**Figure 4:**
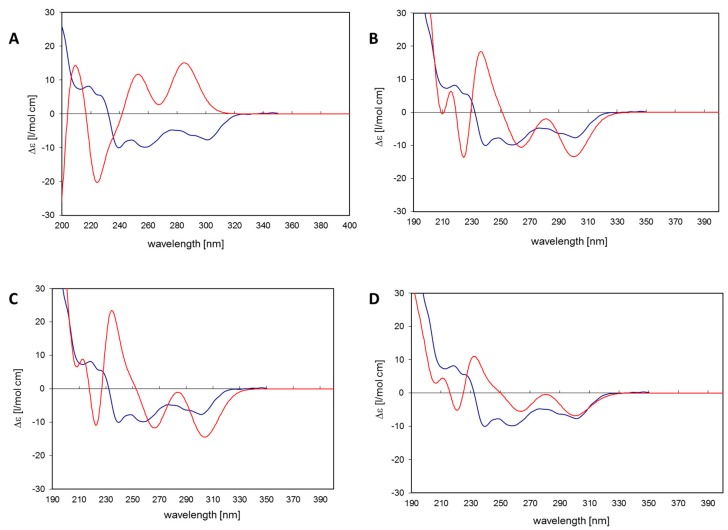
(**A**) Conformer 1: 11*R,Ra* myricanol. **Blue**: Experimental CD spectrum of myricanol. **Red**: CD spectrum simulated for conformer 1 by time-dependent density functional theory (TDDFT): RB3LYP/6-31G(d,p), nstates = 30. No shift, no scaling of calculated spectrum; (**B**) Conformer 2: 11*R,Sa*-myricanol (a). **Blue**: Experimental CD spectrum of myricanol. **Red:** CD spectrum simulated for conformer 2 by TDDFT: RB3LYP/6-31G(d,p), nstates = 30. No shift, no scaling of calculated spectrum; (**C**) Conformer 3: 11*R,Sa*-myricanol (b), conformation corresponds to the X-ray structure. **Blue**: Experimental CD spectrum of myricanol. **Red**: CD spectrum simulated for conformer 3 by TDDFT: RB3LYP/6-31G(d,p), nstates = 30. No shift, no scaling of calculated spectrum. (**D**) **Blue**: Experimental CD spectrum of myricanol. **Red**: Averaged CD spectrum for the *R,Sa* (97%) and *R,Ra* form (3%). TDDFT: RB3LYP/6-31G(d,p), nstates = 30. Calculated spectrum was red-shifted by −0.15 eV and scaled by factor 0.5.

The 11*R*,*Sa* (b) conformation corresponds to the X-ray structure of myricanol determined by Begley et al. [19]. The simulated CD spectrum of the energetically most favorable 11*R*,*Sa*-myricanol (conformer C, Figure 3C) was found to be similar to the experimentally determined spectrum (Figure 4C). The major cotton effects are of equal sign, indicating that the compound indeed has the 11*R*,*Sa*-configuration, as would also be expected based on its reported crystal structure. The spectrum would appear exactly the opposite if the 11*S*,*Ra*-enantiomer was present. The spectrum for the much less favorable 11*R*,*Ra*-atropisomer (conformer 1, Figure 3A) shows almost entirely the opposite sign (Figure 4A). Since its internal energy is predicted to be more than 2 kcal/mol higher than that of conformer C, it would only represent an insignificant fraction in the conformational equilibrium. The two different conformers of *11R*,*Sa* myricanol (2 and 3, Figure 3B,C) show similar signs with the experimental spectrum (Figure 4B,C) which supports the presence of the 11*R*,*Sa*-configuration. The Boltzmann distribution calculated on the basis of the B3LYP/6-31D(d,p) energies of the three conformers would correspond to an even lower amount of conformer 2 (0.3%). Therefore, an averaged spectrum was generated for the theoretical equilibrium mixture corresponding to 97% *R*,*Sa* and 3% *R*,*Ra*-myricanol and compared with the experimental CD spectrum of myricanol (Figure 4D), presenting a very good match with the experimental spectrum. A slight inconsistency observed in the 230 nm range (Figure 4B–D) can be explained by an exchange of two electronic transitions very close to each other, at 217 and 223 nm. However, this does not change the general picture of the main bands at long and short wavelength being of the same sign. According to these calculations, natural myricanol was concluded to be 11*R* and very predominantly *Sa*-configured. This corresponds to the X-ray structure and matches the CD data of myricanol reported in literature [19,30]. It should hence be clear that in the case of the 11*S*-configured compound, the *Ra*-atropisomer (i.e., 11*S*,*Ra*, representing the enantiomer of the depicted structure) would be the energetically most favorable form and the cotton effects in the CD spectrum would show exactly opposite sign. It is possible that a very small amount of the *Ra*-atropisomer is present in the conformational equilibrium. Finally, a confirmation of 11*R*-configuration of diarylheptanoids (**1**–**3**, **7**, **8**, **10**, **12**, **13**, **15**, and **16**) was demonstrated by their very similar experimental CD spectra (Figure 2) showing congruency to that of myricanol. Experimental CD spectrum of myricanol in comparison to averaged CD spectrum for the *S*,*Sa* (87%) and *S*,*Ra* form (13%) can be find in the Supplementary Materials.

## 3. Discussion

A systematic investigation of the diarylheptanoid pattern of *Morella salicifolia* revealed the presence of 16 cyclic diarylheptanoids (*meta*, *meta*-bridged biphenyls), among them six previously unknown compounds. The secondary metabolite pattern of *M. salicifolia* demonstrated the close taxonomic relationship of the new genera *Morella and Myrica* resulting from the taxonomic reorganization of the former postulated genus *Myrica* [1]. The taxonomic relationship was deduced from comparison of isolated known compounds (**7**–**16**) to the same compounds also reported from *Myrica* species [31,32,33]. This makes it probable that cyclic diarylheptanoids are characteristic constituents of both *Morella* and *Myric*a species.

Additionally, a comparison of experimental data with simulation of electronic circular dichroism (ECD) data revealed that there is no effect of an attached glycoside or sulfate group on the natural aglycone absolute configuration. The obtained absolute configuration of natural myricanol in this study matches very well to that achieved by X-ray in the group of Begley [19]. However, in this study the established CD calculation of myricanol was further used as a reference and a non-destructive method to determine the absolute configuration of isolated diarylheptanoids containing glycosides or sulfate groups. The method used is reliable as the obtained results are in agreement with the published crystal structure. It should also be noted that this method was used to determine the absolute configuration of the *meta*,*meta-*bridged biphenyls with chiral center at position 11. There was no sample to prove if the same method can also be applied for the cyclic diarylheptanoids of the *meta*,*para-* diphenyl ether type as well as for the ones without chiral center at position 11.

## 4. Materials and Methods

### 4.1. General Experimental Procedures

Optical rotations were measured in methanol (MeOH for spectroscopy, Merck, Darmstadt, Germany) at 25 °C on a Unipol L 1000 polarimeter (Schmidt and Haensch, Berlin, Germany). CD spectra were recorded on a Jasco J-710 spectrometer (Jasco, Groß-Umstadt, Germany) at a wavelength range of 195–350 nm. UV spectra (MeOH for spectroscopy, Merck, Darmstadt, Germany) were measured using a Cary 50 Scan UV spectrophotometer (Varian, Darmstadt, Germany) equipped with Cary WinUV 3.00 software (Varian, Darmstadt, Germany). The 1D-^1^H, 1D-^13^C, [^1^H-^13^C]-heteronuclear single quantum coherence (HSQC), [^1^H-^13^C]-HMBC, [^1^H-^1^H]-COSY and [^1^H-^1^H]-ROESY NMR experiments were recorded on a Bruker AVANCE 600 spectrometer (600.25 MHz for ^1^H- and 150.93 MHz for ^13^C-NMR (Bruker, Ettlingen, Germany), and referenced to tetramethylsilane (TMS). Samples were dissolved in methanol-*d*_4_ (99.8%, Sigma-Aldrich, Taufkirchen, Germany), acetone-*d*_6_ (99.8%), or pyridine-*d*_5_ (99.5%, both Deutero, Kastellaun, Germany). Low-resolution (LR)-ESI-MS was measured using the TSQ 7000 spectrometer (Thermo Quest, Finnigan, Egelsbach, Germany) and high-resolution (HR)-ESI-MS was measured using the Q-TOF 6540 UHD mass spectrometer (Agilent Technologies, Santa Clara, CA, USA).

MeOH and acetonitril of HPLC grade (Merck, Darmstadt, Germany) were used for chromatography. For extraction and analysis of MeOH, dichloromethane (DCM) and EtOAc of analytical grade (Acros Organics, Morris Plains, NJ, USA) were used. Open column chromatography was performed using Sephadex^®^ LH-20 (25–100 µm, 265 g, 90 × 4.76 cm, GE Healthcare GmbH, München, Germany) and fraction control was done by analytical thin layer chromatography (TLC, silica gel 60 F_254_ aluminium sheets, 20 × 20 cm). The standard mobile phase used for all TLC analyses was ethylacetate/water/acetic acid/formic acid (100 + 26 + 11 + 11). Detection was done at visible light (VIS), and UV 254 and 366 nm before and after spraying a plate with anisaldehyde-sulfuric acid and heating a TLC sheet at 105 °C for 3–10 min.

Centrifugal partition chromatography (CPC) was performed using SPOT centrifugal partition chromatography (Armen Instrument, Saint-Avé, France) with EtOAc (for analysis, Acros Organics) and water as solvent system. Two modes were used; ascending mode, whereby EtOAc was a mobile phase, and descending mode, whereby water was as a mobile phase: rotation = 800 rpm, flow rate 5 mL/min, volume collected in a test tube = 10 mL. Changing from ascending to descending mode was done when no more spots were detected on a TLC sheet when visualized under 254 nm. Final purification of isolated compounds was achieved on semi preparative HPLC using the ProStar HPLC (Varian, Darmstadt, Germany) coupled with the Purospher STAR RP18 column (Eclipse XDB-C18, 250 × 1.4 mm, 5 µm).

### 4.2. Plant Material

The bark of *Morella salicifolia* (Hochst. ex A. Rich.) Verdc. & Polhill was collected in February 2013 at the Monduli mountain ranges in the Arusha region, Tanzania. Identification of the plant was done by Mr. Daniel Sitoni (a senior botanist from the Tanzania National Herbarium (TNH)). Specimen was stored at the TNH with voucher number CK 7792. The collected bark material was spread on a clean cotton cloth under direct sunlight with a temperature between 30 and 35 °C until it was completely dried.

### 4.3. Extraction and Isolation Procedure

Dried pulverized *M. salicifolia* bark (390.1 g) was mixed with 400 g of sea sand, packed in a column, and macerated overnight with 1 L of dichloromethane (DCM). After maceration, bulk extraction of *M. salicifolia* bark was performed using four different solvents with increasing polarity, approximately 4 L each. The solvents used for the extraction were DCM, EtOAc, MeOH 100%, and MeOH 50% (*v*/*v*). The extraction resulted in respective DCM (7.7 g), EtOAc (1.1 g), MeOH (162.1 g) and MeOH 50% (25.9 g) extracts after evaporation using a rotary vacuum evaporator at 40 °C for complete dryness, mixing with water, and complete freezing at −20 °C followed by lyophilization has been done. The dried powder crude extracts were stored at 4 °C in the refrigerator.

MeOH extract (162.1 g) was subjected to Sephadex^®^ LH-20 column chromatography with a 12-g crude portion of extract in each run, aimed at separating tannins from non-tannin compounds (0–1240 mL). Two eluents were used, ethanol 70% (*v*/*v*) and acetone 70% (*v*/*v*). Seven fractions (S1–S7) were obtained. Fractions S1–S6 were eluted with ethanol 70%. Fraction S7, which comprised proanthocyanidins polymers, was retained in the column and could not be eluted with ethanol 70%. Its elution was achieved by acetone 70%. For isolation of diarylheptanoids, fraction S2 (15.7 g, 450–560 mL) was fractionated by means of flash chromatography with the RP-18 pre-packed column eluted with water (A) and MeOH (B), flow rate of 40 mL/min, and a gradient of 20–40% B (10 min) → 40–100% B (20 min) → 100% B (30 min). Seven fractions (F1–F7) were obtained. The gradient starting at polar conditions resulted in elution of most of the sugars at the beginning of the chromatography (fraction F1), and hence their separation from other compounds.

F5 (1.44 g, 851–920 mL) was fractionated by CPC (EtOAC/water system, flow = 5 mL/min, 800 rpm) and resulted in nine fractions (F5.C1–F5.C9). F5.C1 (79.5 mg, 101–160 mL) was subjected to semi-preparative HPLC eluted with water (A) and MeOH (B), flow rate 3 mL/min, gradient 50–70% B (20 min) → 70–100% B (0.1 min) → 100% B. Three peaks were eluted, whereby two peaks resulted from the isolation of compounds **14** (3.1 mg, *t*_R_ = ~12 min), and **10** (6.5 mg, *t*_R_ = ~13.3 min). The third peak at *t*_R_ = ~14 min (42.9 mg) was not pure, and was hence subjected to a subsequent purification step with flash chromatography (column: silica gel Reveleris Flash Cartridges, 20 µm, 12 g) eluted with CHCl_3_ (A) and MeOH (B), flow rate: 15 mL/ min, gradient: 5% B (40 min) → 5–100% B (25 min) → 100% B (15 min), and resulted in isolation of compounds **11** (10.2 mg, *t*_R_ = 6 min) and **13** (13.3 mg, *t*_R_ =9 min). Similarly, separation and purification of F5.C3 (21.8 mg, 281–480 mL) using preparative HPLC with the same procedure as for purification of F5.C1 led to the isolation of compounds **3** (0.7 mg, *t*_R_ = ~7.5 min), **2** (0.6 mg, *t*_R_ = ~5.8 min), and **10** (1.81 mg). Further separation and purification of F5.C4 (22.7 mg, 481–810 mL) by preparative HPLC eluted with water + 0.02% trifluoroacetic acid (TFA) (A), and MeCH + 0.02% TFA (B), flow rate: 2 mL/min, gradient: 25–35% B (30 min) → 35–65% B (1 min) → 65% B (4 min), resulted in compound **6** (0.9 mg, *t*_R_ = ~24.2 min).

Furthermore, purification of F5.C5, (15.5 mg, 811–1330 mL) by RP-18 (semi preparative), eluted with H_2_O (A) and MeOH (B), flow rate: 3 mL/min, gradient: 50–60% B (15 min) → 60–100% B (1 min) → 100% B (4 min), led to isolation of compound **1** (2.5 mg, *t*_R_ = ~10.5 min) and **15** (2.2 mg, *t*_R_ = ~14 min). F5.C7 (39.5 mg, 1140–1160 mL) and F5.C8 (11.0 mg, 1161–1200 mL) were separated using the same procedure as for F5.C5 and resulted in compounds **7** (1.0 mg, *t*_R_ = ~6.3 min), **16** (2.6 mg, *t*_R_ = ~11.9 min) and **4** (1.0 mg, *t*_R_ = ~10.5 min) for F5.C7. F5.C8 resulted in the reisolation of **3** (1.8 mg, *t*_R_ = ~12 min) and **4** (4.2 mg, *t*_R_ = ~10.5 min) and compound **5** (0.9 mg, *t*_R_ = ~17.1 min).

F6 (from S2) was fractionated using flash chromatography (column: silica gel Reveleris Flash Cartridges, 20 µm, 12 g), eluted with CHCl_3_ (A) and MeOH (B), flow: 15 mL/min, gradient: 5–12% B (30 min) → 12–60% B (1 min) → 60–100% B (9 min) → 100% B (10 min), and resulted in the 10 subfractions F6.1–F6.10. Subfraction F6.3 (43.4 mg, 21–40 mL) was subjected to preparative RP-18 (semi-preparative) HPLC, eluted with H_2_O (A) and MeOH (B), flow rate 2 mL/min, gradient: 75–80% B (15 min) → 80–100% B (1 min) → 100% B (3 min), and led to the isolation of compounds **8** (30.5 mg, *t*_R_ = ~10.5 min) and **9** (6.6 mg, *t*_R_ = ~11.8 min). F6.5 (1.8 mg, 96–140 mL) was further purified using RP-18 semi-preparative HPLC eluted with water (A) and MeOH (B), flow 2 mL/min, gradient: 70% B (15 min) → 70–100% B (1 min) → 100% B (4 min), and resulted in the isolation of compound **12** (0.7 mg, *t*_R_ = ~11 min).

*Salicimeckol* (**1**). White, amorphous powder; ${\left[\alpha \right]}_{D}^{25}$ −56 (*c* 0.1, MeOH); UV λ_max_ (MeOH) (log Ɛ) 213 (3.90), 250 (3.88), and 295 (3.67) nm; for ^1^H- and ^13^C-NMR data see Table 1 and Table 2; HR-ESI-MS *m*/*z* 535.2185 [M − H]^−^ (calculated for C_27_H_35_O_11_ 535.2185).

*Salicireneol A* (**2**). White, amorphous powder; ${\left[\alpha \right]}_{D}^{25}$ −49.0 (*c* 0.1, MeOH); UV λ_max_ (MeOH) (log Ɛ) 214 (4.3), 254 (4.02), and 285 (3.91) nm; for ^1^H- and ^13^C-NMR data see Table 1 and Table 2; HR-ESI-MS *m*/*z* 489.2137 [M − H]^−^ (calculated for C_26_H_33_O_9_ 489.2130).

*Salicireneol B* (**3**). White, amorphous powder; ${\left[\alpha \right]}_{D}^{25}$ −33.6 (*c* 0.1, MeOH); UV λ_max_ (MeOH) (log Ɛ) 216 (4.11), 253 (4.0), and 298 (4.04) nm; for ^1^H- and ^13^C-NMR data see Table 1 and Table 2; HR-ESI-MS *m*/*z* 505.2078 [M − H]^−^ (calculated for C_26_H_33_O_10_ 505.2079).

*Saliciclaireone A* (**4**). White, amorphous powder; ${\left[\alpha \right]}_{D}^{25}$ −51.6 (*c* 0.1, MeOH); UV λ_max_ (MeOH) (log Ɛ) 214 (4.21), 250 (4.10), and 300 (4.04) nm; for ^1^H- and ^13^C-NMR data see Table 1 and Table 2; HR-ESI-MS *m*/*z* 679.2682 [M − H]^−^ (calculated for C_33_H_43_O_15_ 679.2607).

*Saliciclaireone B* (**5**). White, amorphous powder; ${\left[\alpha \right]}_{D}^{25}$ −43.1 (*c* 0.1, MeOH); UV λ_max_ (MeOH) (log Ɛ) 214 (4.08), 250 (4.09), and 298 (4.03) nm; for ^1^H- and ^13^C-NMR data see Table 1 and Table 2; HR-ESI-MS *m*/*z* 679.2682 [M − H]^−^ (calculated for C_33_H_43_O_15_ 679.2607).

*Saliciclaireone C* (**6**). Off-white, amorphous powder; ${\left[\alpha \right]}_{D}^{25}$ −36 (*c* 0.1, MeOH); UV λ_max_ (MeOH) (log Ɛ) 215 (4.06), 252 (4.03), and 295 (3.99) nm; for ^1^H- and ^13^C-NMR data see Table 1 and Table 2; HR-ESI-MS *m*/*z* 649.2509 [M − H]^−^ (calculated for C_32_H_41_O_14_ 649.2502).

### 4.4. Determination of Absolute Configuration of Glycosides

Determination of the glycosides and their absolute configuration was achieved by recording the ^1^H-NMR spectra of the per-*O*-(*S*)-2-methylbutyrate (SMB) derivatives and comparison with the ^1^H-NMR of the SMB derivatives of reference sugars [34]. The reference sugars used in this study were d- and l-glucose, d- and l-arabinose, and d- and l-xylose. A quantity of 1 mg glycoside was hydrolyzed using 200 µL of 2 M trifluoroacetic acid (TFA) at 121 °C for 90 min in a Wheaton vials sealed with Teflon-lined screw cap. After hydrolysis, the solvent was evaporated to complete dryness under nitrogen stream. Then, 100 µL of (*S*)-(+)-2-methylbutyric anhydride and 100 µL of pyridine were added to the mixture and incubated at 121 °C for 4 h. The mixture was dried under nitrogen stream for about 8 h and 300 µL of toluene was added to the residue and evaporated. The residue was dissolved in 1 mL of DCM and extracted three times with 2 mL of 2 M Na_2_CO_3_ solution and once with 2 mL H_2_O. The DCM phase containing the SMB derivatives was concentrated using nitrogen stream, then completely dried by the addition of 300 µL of 2-propanol and evaporation. Preparation of samples for NMR measurement was done by dissolving each of the obtained SMB derivatives in 0.6 mL of deuterated acetone. The mixture was transferred to the NMR tube and their ^1^H-NMR spectra were recorded at 300 MHz, 298 K. The same procedure was applied to reference monosaccharides with the exception of hydrolysis reaction. Absolute configuration and type of glycoside was confirmed by comparing chemical shifts and coupling constants of anomeric proton resonances of the SMB derivative to that of reference monosaccharides SMB derivative. For compounds with two sugar substitutions, ^1^H-NMR of SMB derivatives were compared to a ^1^H-NMR of the SMB derivative of a mixture of the two concerning reference sugars.

### 4.5. Enzymatic Deglycosidation of Cyclic Diarylheptanoid Glycosides

Enzymatic hydrolysis was done using β-glucosidase (almond) to obtain the aglycone by deglycosidation of cyclic diarylheptanoid glycosides [20] with an exception for compounds **12** and **15**. The enzyme hydrolysis was conducted as follows: 21.0 mg (0.040 mmol) of a compound was dissolved in 3.0 mL of 0.2 M acetate buffer (0.2 M acetic acid + 0.2 M sodium acetate, pH 4.4). The solution was treated with 40 mg of β-glucosidase and stirred. The mixture was incubated while stirring in the ultrasonic water bath at 38 °C for 2 days. After incubation, the reaction mixture was mixed with 10 mL of absolute EtOH and evaporated to dryness by using a vacuum rotary evaporator at 40 °C. The residues were dissolved in CHCl_3_/H_2_O (1 + 1), thoroughly mixed, left to settle and finally the two phases were separated. The obtained upper and lower phase were dried and separately purified by semi-preparative HPLC method eluted with water (A) and MeOH (B), flow rate: 2 mL/min, gradient: 60% B (10 min) 60–100% B (1 min) → 100% B (4 min). The collected peaks from HPLC purification of the upper and lower phase were dried under nitrogen stream and subjected to ^1^H-NMR measurement. Prior to ^1^H-NMR measurement, the samples were dissolved in 0.6 mL deuterated methanol, filled in the NMR tubes followed by measurement at 300 MHz, 298 K. The recorded ^1^H-NMR spectra data of the obtained peaks were compared with the existing data to identify which peak stands particularly for the aglycone.

### 4.6. Circular dichroism Spectra: Measurement and Simulation

A quantity of 0.6 mg of the isolated compounds and the obtained aglycone from enzymatic deglycosydation were dissolved in 10 mL methanol, and their CD spectra were recorded. Recorded CD spectra data were used during electronic CD spectra simulation using time-dependent density functional theory (TDDFT) quantum mechanics. 

Molecular models of the 11*R*-enantiomer of myricanol were generated with the software package Molecular Operating Environment (MOE, CCG, Montréal, Canada). After a low-mode-dynamics conformational search (default settings), three conformations were obtained within an energy window of 3 kcal/mol of which conformer 1 corresponded to the 11*R*,*Ra* and conformers 2 and 3 to two slightly different 11*R*,*Sa* forms. The 3D structures were exported to the software Gaussian 03W and completely energy-minimized using the B3LYP density functional and the 6-31D (d,p) basis set. According to these calculations, conformer 3 was the energetically most favorable form: conformer 1 had a value of 2.01, and conformer 2 was even 3.31 kcal/mol higher in energy. The Boltzmann distribution calculated with these energy differences indicates that conformer 3 would strongly dominate (97%) in a conformational equilibrium, while only 2.7% and 0.3% would be contributed by conformers 1 (11*R*,*Ra*) and 2 (11*R*,*Sa*), respectively. The geometry of the most favorable conformer 3 is in very good agreement with the crystal structure of myricanol, as published by Begley et al. [19]. Electronic CD spectra were simulated for all three conformers by performing a time-dependent DFT (TDDFT) calculation for the first 30 electronic transitions of each of the three conformers using the same basis set as mentioned above. The resulting transition vectors (R, length) were used to simulate the CD spectra for each form by multiplying them with Gaussian functions of width of 0.1 eV and summing the resulting curves up over the whole energy/wavelength scale. Furthermore, an averaged spectrum was generated for the theoretical equilibrium mixture corresponding to 97% *R*,*Sa* (conformer 3) and 3% *R*,*Ra*-myricanol (conformer 1). The resulting spectra were compared with the experimental CD spectrum of myricanol and the obtained results were used to confirm the absolute configuration of the aglycone myricanol and other isolated diarylheptanoids.

## Figures and Tables

**Table 1:** ^1^H-Nuclear magnetic resonance (NMR) data of **1**–**6** (600 MHz, methanol-*d*_4_, 298 K, δ ppm, mult, *J*, Hz).

Number	1	2	3	4	5	6
5		6.85 brs	7.04 brs			
7	4.92 dd (3.6, 11.4)	2.98 m 2.54 m	2.78 m 2.64 m	2.91 m * 2.78 m *	3.19 m 2.97 m	2.73 m 2.96 m
8	2.13 m 1.97 m	1.97 m 1.83 m *	1.74 m 1.59 m	1.89 m * 1.89 m *	2.87 m * 2.87 m *	1.90 m * 1.90 m *
9	1.52 m * 1.15 m	1.61 m 1.43 m	1.27 m * 1.03 m	1.70 m * 1.70 m *		1.74 m * 1.74 m *
10	1.79 m 1.53 m *	1.83 m * 1.52 m	1.27 m * 0.90 m	2.78 m * 2.65 m *	2.75 m * 2.64 m *	2.80 m * 2.61 m
11	3.84 m	3.91 m	3.05 m		1.74 m * (2H)	
12	2.13 m 1.63 m	2.23 m 1.63 m	1.43 m (2H)	2.91 m * (2H)	1.97 m * (2H)	2.79 m * 2.93 m *
13	2.84 m * 2.84 m *	2.85 m 2.01 m	2.64 m * 2.60 m *	2.78 m *	2.75 m * 2.64 m *	2.95 m * 2.95 m *
15	7.02 dd (2.2, 8.2)	7.04 dd (2.3, 8.2)	5.66 brs	7.05 dd (2.1, 8.2)	7.00 dd (2.2, 8.2)	7.03 dd (2.9, 8.7)
16	6.77 d (8.2)	6.78 d (8.2)		6.80 d (8.2)	6.82 d (8.2)	6.78 d (8.2)
18	6.87 brs	7.07 brs	6.64 brs	6.64 brs	6.87 d (1.9)	6.67 d (2.3)
19	6.87 s	6.76 brs	6.87 brs	6.57 s	6.42 s	6.58 s
20	3.95 s	3.88 s	3.80 s	3.94 s	3.84 s	3.93 s
21	3.95 s			3.80 s	3.95 s	3.81 s
1’	5.05 d (7.4)	4.99 d (7.3)	5.00 d (7.6)	4.99 d (7.2)	4.88 d (7.6)	4.97 d (7.2)
2’	3.50 m *	3.38 m *	3.54 m *	3.50 m *	3.52 m *	3.44 m *
3’	3.39 m *	3.20 m *	3.49 m *	3.50 m *	3.39 m *	3.43 m *
4’	3.47 m *	3.07 m	3.42 m *	3.50 m *	3.53 m *	3.44 m *
5’	3.32 m *	3.29 m *	3.42 m *	3.41 m	3.46 m	3.38 m
6’	3.80 dd (2.4, 11.8) 3.64 dd (5.7, 11.8)	3.45 m * 3.45 m *	3.89 brd (12.3) 3.71 (dd, 4.0, 1.8)	4.08 dd (1.8, 11.5) 3.78 m	4.11 dd (2.2, 11.2) 3.79 m	3.96 dd (2.1, 11.0) 3.56 m *
1”				4.28 d (7.8)	4.25 m	4.83 brs
2”				3.18 m	3.24 m *	3.91 m
3”				3.30 m *	3.22 m *	3.78 m *
4”				3.25 m *	3.27 m *	3.83 m *
5”				3.25 m *	3.27 m *	3.67 dd (3.2, 11.7) 3.58 m *
6”				3.84 m 3.63 dd (5.2, 11.9)	3.81 dd (2.5, 11.9) dd (5.4, 11.9)	

* Overlapping signals.

**Table 2:** ^13^C-NMR data of **1**–**6** (150 MHz, methanol-*d*_4_, 298 K, δ ppm).

Number	1	2	3	4	5	6
1	126.8	128.0	126.2	126.7	125.4	126.5
2	120.2	128.2	131.5	129.4	129.6	129.6
3	147.0	140.9	144.8	146.6	148.3	146.5
4	152.2	152.7	154.0	149.2	146.3	148.9
5	150.0	113.6	115.3	149.8	148.7	149.9
6	131.0	131.8	137.9	131.8	131.9	127.6
7	66.7	31.6	36.2	28.4	23.6	28.2
8	35.6	27.5	31.2	25.7	41.8	25.6
9	21.8	23.8	23.4	22.9	215.8	22.9
10	40.0	40.2	39.5	46.2	44.6	45.8
11	68.9	69.1	72.2	217.2	22.7	216.1
12	35.5	35.5	37.4	43.1	26.2	42.7
13	27.7	27.9	29.3	29.1	31.8	28.7
14	131.3	132.0	136.3	133.2	132.1	132.8
15	131.0	130.6	115.2	130.2	130.9	129.8
16	117.1	117.2	151.6	117.5	117.4	117.1
17	153.1	152.1	144.7	152.8	152.2	152.7
18	135.8	135.0	122.7	133.9	133.7	133.8
19	130.8	127.6	124.2	130.0	129.5	129.7
20	61.1	56.6	56.4	62.3	62.1	61.8
21	61.9			61.8	62.2	61.6
1’	104.8	104.8	102.9	105.0	105.2	104.9
2’	75.5	75.3	74.7	75.4	74.8	75.3
3’	71.4	71.1	77.8	77.0	76.3	77.5
4’	77.7	77.5	71.1	71.0	70.5	75.3
5’	78.5	78.6	78.0	77.5	77.0	76.6
6’	62.1	62.6	62.3	69.5	69.3	68.0
1”				104.4	103.8	109.6
2”				75.0	74.3	82.8
3”				77.7	77.0	78.6
4”				71.4	70.8	85.6
5”				77.7	77.2	62.7
6”				62.6	62.2	

## Supplementary Materials

Supplementary materials are available online.
