# Supplementary material for: Diarylheptanoid Glycosides of Morella salicifolia Bark
Source: Molecules. 2017 Dec 19;22(12):2266. doi: 10.3390/molecules22122266 (PMC6149793; doi:10.3390/molecules22122266)
Supplement: Supplementary file 1 [file molecules-22-02266-s001.pdf]

molecules

MDPI

Article

Diarylheptanoid Glycosides of *Morella salicifolia* Bark

Edna Makule [1,2], Thomas J. Schmidt [3], Jörg Heilmann [1,*] and Birgit Kraus [1,*]

Supplementary Material

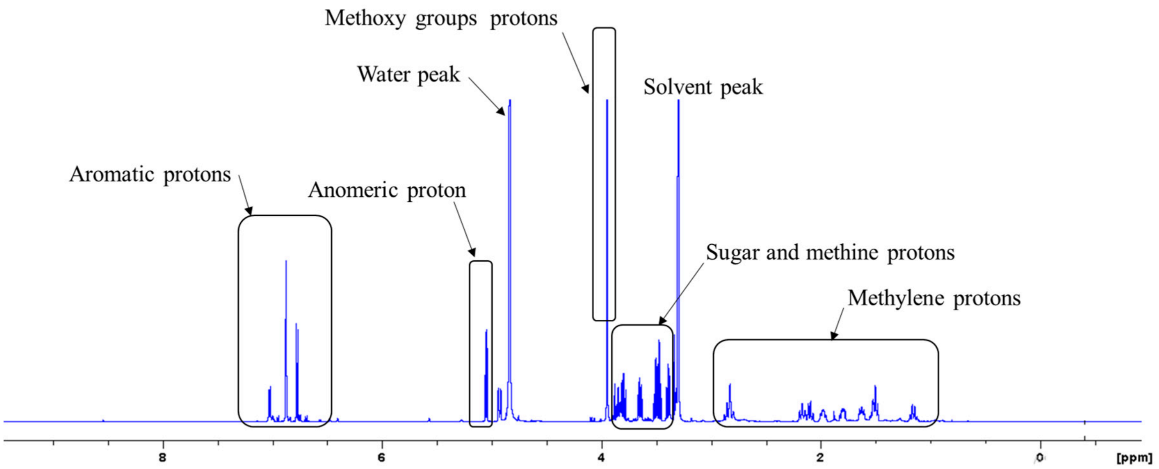

Figure S1. ^{1}H NMR spectrum (600 MHz, methanol-d$_4$, 298 K) of compound **1**: salicimeckol (7-hydroxymyricanol 5-*O*-*β*-D-glucopyranoside).

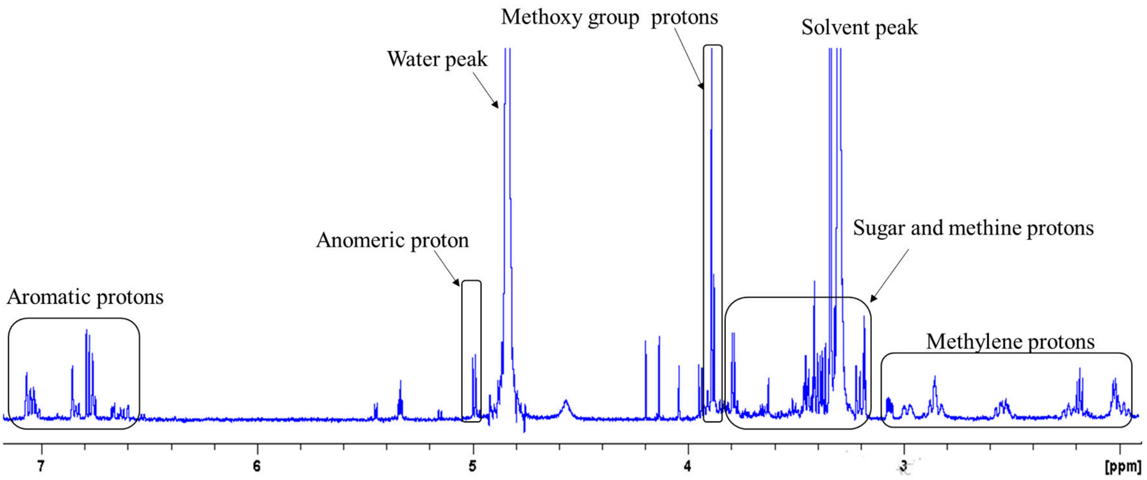

Figure S2. ^{1}H-NMR spectrum (600 MHz, methanol-d$_4$, 298 K) of compound **2**: salicireneol A (juglanin B 3-*O*-*β*-D-glucopyranoside).

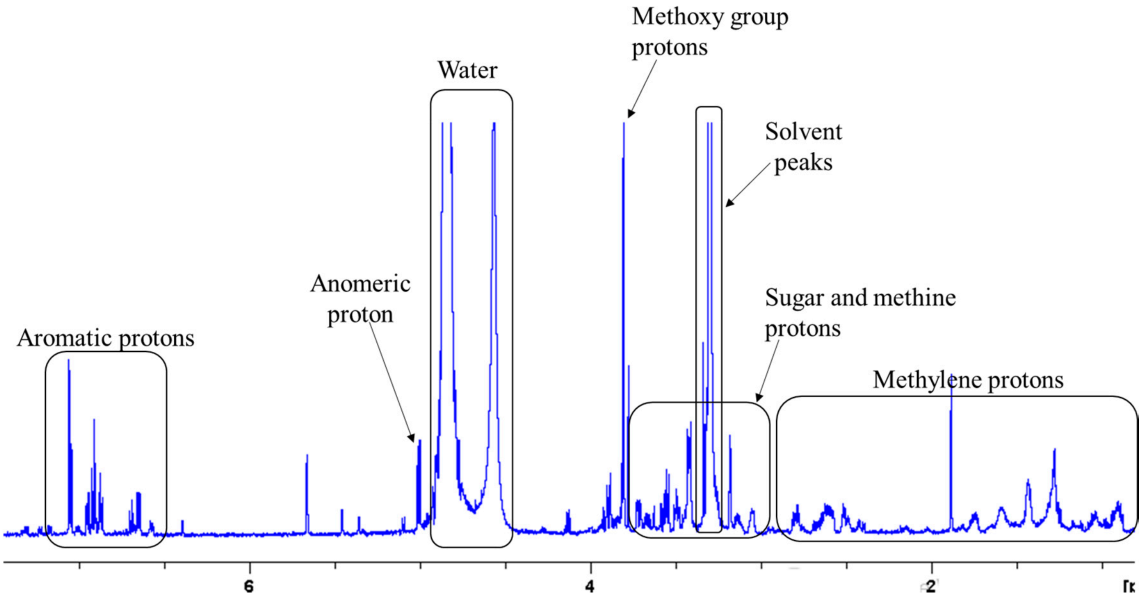

Figure S3. ^{1}H-NMR spectrum (600 MHz, methanol-d$_4$, 298 K) of compound **3**: salicireneol B (16-hydroxyjuglanin B 17-O-*β*-D-glucopyranoside).

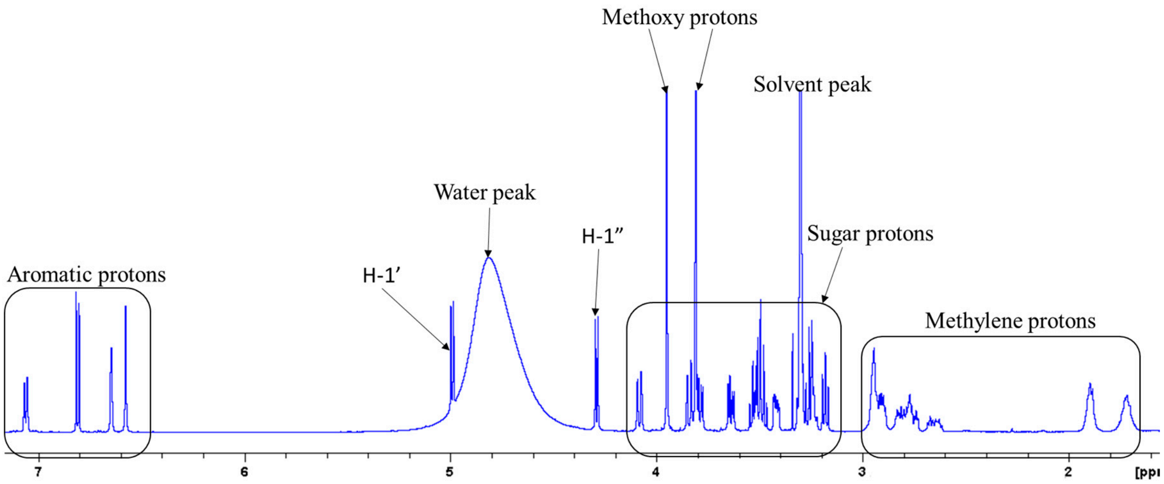

Figure S4. ^{1}H-NMR spectrum (600 MHz, methanol-d$_4$, 298 K) of compound **4**: saliciclaireone A (myricanone 5-*O*-*β*-D-glucopranosyl-(1-6)-*β*-D-glucopyranoside).

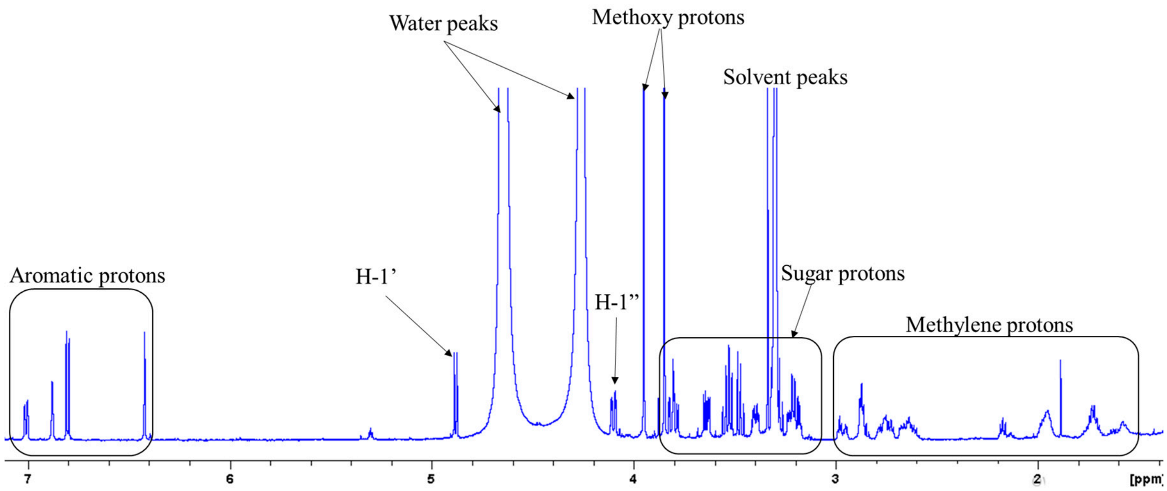

Figure S5. ^{1}H-NMR spectrum (600 MHz, methanol-d$_4$, 298 K) of compound **5**: saliciclaireone B (neomyricanone 5-*O*-*β*-D-glucopranosyl-(1-6)-*β*- D-glucopyranoside).

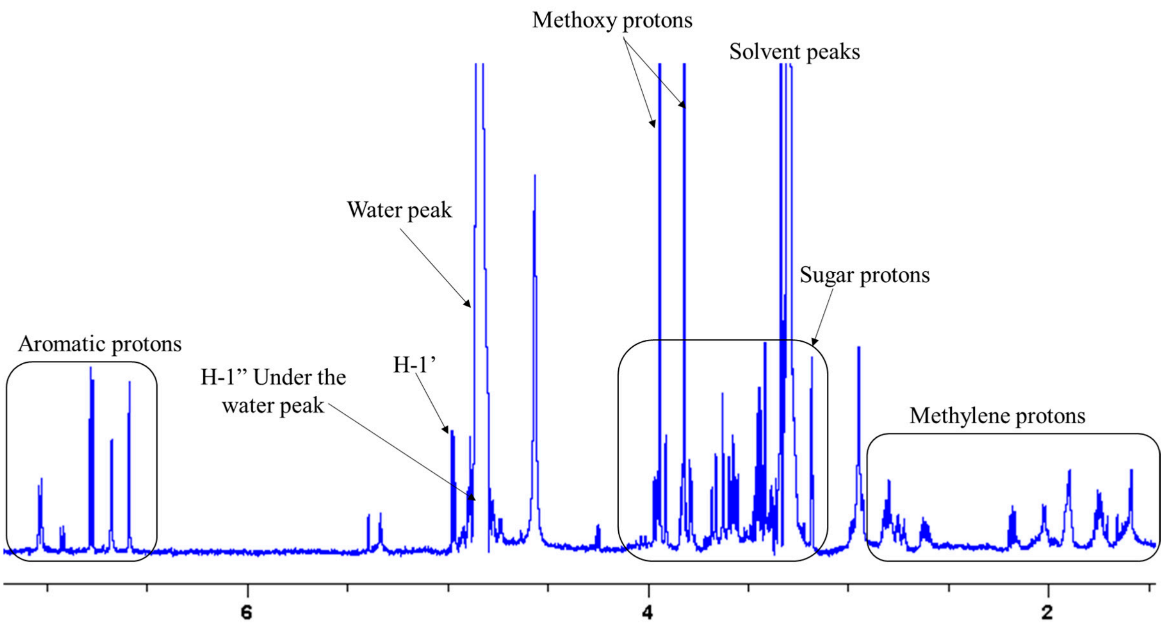

Figure S6. ^{1}H-NMR spectrum (600 MHz, methanol-d$_4$, 298 K) of compound **6**: saliciclaireone C (myricanone 17-*O*-*α*-L-arabinofuranosyl-(1-6)-*β*-D-glucopyranoside).

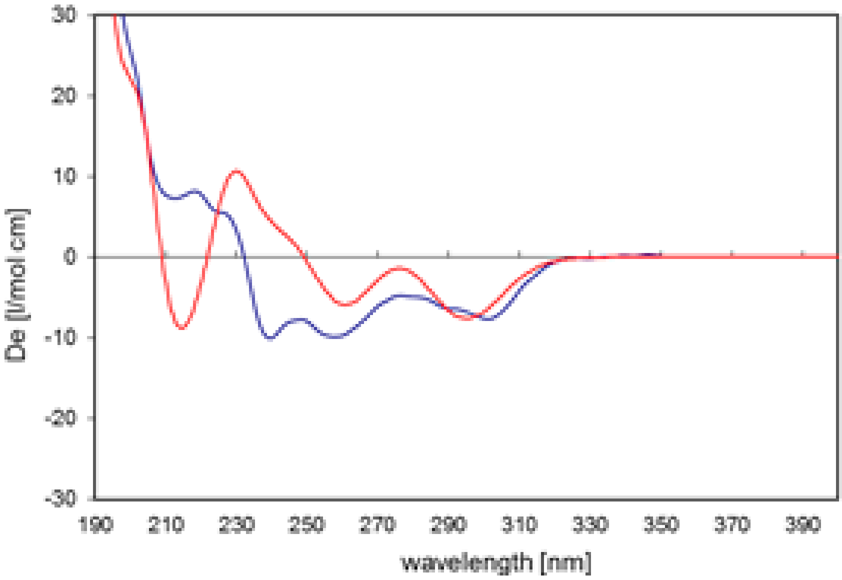

Figure S7. Blue: Experimental CD spectrum of myricanol. **Red:** Averaged CD spectrum for the *S*,*Sa* (87%) and *S*,*Ra* form (13%). TDDFT: RB3LYP/6-31G(d,p), nstates = 30. Calculated spectrum was red-shifted by −0.15 eV and scaled by a factor of 0.67.
